# Proteomics Analysis of Plasma Biomarker of Cognitive Frailty in Elders Who Locally Reside in Chiang Mai Province of Thailand

**DOI:** 10.3390/life15081330

**Published:** 2025-08-21

**Authors:** Paitoon Aobchey, Parunya Chaiyawat, Mathuramat Seesen, Jetsada Ruangsuriya

**Affiliations:** 1 Functional Food Research Center for Well-Being, The Multidisciplinary Research Institute, Chiang Mai University, Chiang Mai 50200, Thailand; paitoon.aob@cmu.ac.th; 2 Center of Multidisciplinary Technology for Advanced Medicine, Faculty of Medicine, Chiang Mai University, Chiang Mai 50200, Thailand; parunya.chaiyawat@cmu.ac.th; 3 Department of Community Medicine, Faculty of Medicine, Chiang Mai University, Chiang Mai 50200, Thailand; mathuramat.s@cmu.ac.th; 4 Department of Biochemistry, Faculty of Medicine, Chiang Mai University, Chiang Mai 50200, Thailand

**Keywords:** plasma biomarker, 2D gel electrophoresis, proteomic approach, fibrinogen gamma chain

## Abstract

Cognitive frailty in elders has become very common in Thailand society and is extremely difficult to manage in clinical practice due to poor detection and diagnosis. Plasma proteomic studies may be able to provide promising protein markers specific to this condition in order to improve the quality of life in the elderly. The purpose of this study was to differentiate and identify a potential protein marker of cognitive frailty in plasma samples by proteomic approaches. Nine plasma samples from elders with cognitive frailty or non-frailty were pooled and subjected to 2D gel separation. Conventional analysis was performed with the stained gel, and the selected protein spot was identified by liquid chromatography–tandem mass spectrometry coupled to a SCIEX TripleTOF 6600+. It was found that fibrinogen gamma (FGG) chain protein was suggested. FGG was further validated with a commercial ELISA kit using both pooled and individual plasma samples. It was found that both pooled and individual samples showed a significant reduction in FGG levels in elders with cognitive frailty. The results from this study suggest that FGG chain may be a potentially useful plasma biomarker for early detection, diagnosis, and therapeutic applications of cognitive frailty in elders in order to achieve sustainable health in an aging society.

## 1. Introduction

An aging society has become the forefront of management policy in all countries around the world, especially regarding healthcare for elders, for whom a huge budget must be allocated to ensure their well-being. It is projected that the number of people aged 60 years or over worldwide will grow by 56 percent, from 901 million to 1.4 billion, between 2025 and 2030. By 2050, the global population of older persons is expected to more than double, reaching nearly 2.1 billion. Moreover, the number of people aged 80 years or over, the “oldest-old” persons, is increasing even faster than the overall older population [1]. Since 2005, Thailand has entered an aging society, with the proportion accounting for 14.57%, and in 2020, a completely aging society (20%) was officially announced [2]. The proportion of the late-elderly population (aged 80 years and above) shows a clear increasing trend. This has impacted the prevalence of chronic diseases, requiring increased long-term care and higher associated costs, and also affected socioeconomic conditions, particularly related to healthcare. The number of workers to support the aging population is decreasing; in Thailand, only two people of working age are available to care for each elderly person [3].

Due to increased vulnerability in all aspects among elders, health promotion and disease prevention in the elderly population have become serious issues for governments worldwide. It was found that 69.3% of the population aged 60–69 years had chronic diseases, and the prevalence increased to 83.3% in the age group of 80 years and over. For example, joint pain and chronic back pain have a prevalence of up to 1 in 3 other health problems [4], and the prevalence of neurodegenerative diseases like dementia in the elderly was found to be 12.4% along with common clinical syndromes, which contribute to frailty [5]. Frailty is associated with increasing age and chronic diseases and is a major cause of disability, risk of falls, and burden for families and communities. Therefore, promoting and maintaining the health of the elderly are necessary to improve their quality of life and foster a sustainable community in an aging society [6].

Frailty is defined as an impaired ability to endure and respond to stress and can be determined using five phenotypic indicators: unintentional weight loss, slow gait speed, weakened grip strength, exhaustion, and physical inactivity [4]. It was found that 10.7% of adults aged 65–74 years are frail [7]. As mentioned above, frail older adults face higher risks of disabilities, incidents of falls, hospitalization, and death [8]. Clinically, physical frailty is a syndrome with multiple causes and contributing factors characterized by multisystem impairment that increases vulnerability to stress while decreasing physiological reserve [4]. Various methods have been proposed to evaluate frailty, including assessments of physical function, cognition, activities of daily living, comorbidities, deficits, and physiological dysfunctions. Nevertheless, the most widely accepted diagnostic method for frailty relies on the presence of three or more of the five aforementioned indicators, known as Fried’s criteria [4].

Cognitive frailty, which can potentially lead to other neurodegenerative diseases if left untreated, is a geriatric clinical syndrome characterized by the coexistence of mild cognitive impairment (MCI) and physical frailty in the absence of diagnosed dementia [9]. Epidemiological data from Asian populations indicate prevalence rates of 2.7% in Japan and 1.6% in Singapore [10,11], whereas higher rates of approximately 4.4% have been reported in Western countries [12]. Although relatively uncommon in community-dwelling older adults, cognitive frailty has been consistently associated with adverse health outcomes, including increased risks of disability, reduced health-related quality of life, falls, and death [13,14,15]. As a potentially reversible condition involving both physical and cognitive decline, early detection and targeted interventions for modifiable risk factors are critical to mitigating the risk of dementia and preserving functional capacity in older adults.

Using proteomics techniques to study differences in protein expression patterns in cognitive frailty among elder adults allows for the rapid identification of potential plasma biomarkers due to the capability to detect slight changes between groups [16]. The proteomic approach can also facilitate the creation of specific diagnostic models and treatment modalities to improve the quality of life in the elderly. However, specific molecular biomarkers for diagnosis and therapeutic targets of cognitive frailty, an early condition leading to other neurodegenerative diseases, are still lacking.

In this study, we aimed to comparatively identify plasma protein expression of elderly people with cognitive frailty using proteomic approaches. The results suggested in this study could be further developed as a plasma biomarker for the early detection and diagnosis of cognitive frailty in older adults. Ultimately, this will contribute to building a sustainably healthy community in an aging society.

## 2. Materials and Methods

### 2.1. Participants

Participants in this study were randomly selected from groups as reported by Seesen and colleagues in 2021 [17]. With the incorporation of Khua Mung Health Center in Saraphi District, Chiang Mai Province, Thailand, this research was made possible. The study was advertised through the staff at the health center and was opened to all participants who permanently resided in this district and were aged 65–84 years. There were 934 people eligible for this study. After primary screening by the volunteer health center staff to exclude individuals with neurodegenerative diseases such as dementia, psychological conditions such as depression, kidney diseases, hepatic diseases, autoimmune diseases, cancer, acute trauma or illness, and those taking steroidal drugs, 494 adults were selected, and 24 people declined to participate. All participants were assessed for physical frailty using Fried’s criteria [4] and for cognitive impairment using the Thai version of The Montreal Cognitive Assessment-Basic (MoCA-B) [17]. Finally, there were 48 robust and 135 cognitively frail individuals. According to Seesen and colleagues’ observation, 61 cognitively frail people were randomly selected for biochemical analyses, while 9 people were randomly sampled in this study based on the prevalence (13%) in the studied population. Therefore, 9 robust (non-frailty) and 9 cognitive frailty individuals were randomly selected. The Committee for Human Research Ethics of the Faculty of Medicine, Chiang Mai University granted permission to conduct this research under the approval number COA 050/2022.

### 2.2. Plasma Sample Collection and Protein Preparation

A total of 5 mL of fasting blood was drawn from each participant in a heparin-coated tube. A total of 1 mL of whole blood was separated by centrifugation at 3000× *g* for 10 min, and 0.2 mL of plasma was subsequently obtained. The plasma samples were stored at −80 °C for further analyses. Nine plasma samples from each group were pooled by mixing 10 μL from each individual plasma.

Protein quantification was performed using the Pierce Coomassie (Bradford) Protein Assay Kit Prod# 23236 (Thermo Scientific, Waltham, MA, USA) by adding 100× diluted plasma sample into 200 μL of working Bradford solution. The protein concentration was calculated using a standard curve prepared with Pierce Albumin Standard Ampules, 2 mg/mL Prod# 23209 (Thermo Scientific, Waltham, MA, USA).

### 2.3. Two-Dimensional Polyacrylamide Gel Electrophoresis or 2-DE

A total of 500 μg of protein was added to immobilized pH gradient (IPG) buffer (0.5%, pH 4–7) in 65 mM dithioerythritol (DTE) and heated at 95 °C for 5 min. Each sample was mixed with a lysis buffer containing 7 M urea, 4% 3-[(3-cholamidopropyl) dimethylammonio]-1-propanesulfonate) (CHAP), 2 M thiourea, and 0.02% bromophenol blue (sodium salt) in a total volume of 350 μL. After incubation at room temperature (RT) for at least one hour, centrifugation at 12,000× *g* for 20 min was performed. The whole mixture was loaded onto dry IPG strips (Immobiline DryStrip, 18 cm, pH 4–7, GE Healthcare, Chicago, IL, USA). The first-dimension separation was performed using an IPGphore system (Ettan IPGphor3, GE Healthcare, Uppsala, Sweden) under the following conditions. IPG strip rehydration was conducted at 50 V and 25 °C for 14 h. After rehydration, isoelectric focusing (IEF) was performed sequentially at 100 V (1 h), 250 V (1 h), 500 V (1 h), 1000 V (1 h), 3000 V (1 h), and 8000 V until 65 kVh was reached.

Afterward, the IPG strips were equilibrated in equilibrating buffer containing 50 mM Tris-HCl, pH 8.8, 6 M Urea, 30% glycerol, 2% (*w*/*v*) DTE, and a trace of bromophenol blue for 15 min. Subsequently, the equilibrated strip was treated with 2.5% (*w*/*v*) iodoacetamide (IAA) in the equilibrating buffer for 15 min.

Second dimension separation was then performed by placing the IPG strip on the top of the 12.5% polyacrylamide gel (18 × 18 cm) and covering it with 0.5% agarose. The separation was carried out at 45 mA per gel, and the gel was fixed with 10% methanol and 7% acetic acid solution for 3 h at RT. Then, the gel was washed three times in deionized water (dH_2_O) for 15 min each. The washed gel was stained with SYPRO^®^ Ruby gel stain for one hour and washed three more times in dH_2_O for 15 min each. Finally, the gel was scanned with a Typhoon TRIO image scanner (GE Healthcare, Chicago, IL, USA) and analyzed using conventional methods [18].

### 2.4. In-Gel Tryptic Digestion

After the selection of protein spots, proteins in each gel spot were completely destained by 50% acetonitrile (ACN) in 10 mM ammonium bicarbonate (Sigma-Aldrich, Saint Louis, MO, USA). The excised protein spot was treated with 4 mM dithiothreitol (DTT) at 65 °C for 30 min, followed by treatment with 50 mM IAA (Sigma-Aldrich, Saint Louis, MO, USA) at RT with light protection for 30 min. Then, 4 mM DTT was added to stop the reaction by incubation at RT for 5 min. All solutions were removed, and gel pieces were dehydrated by adding 100% ACN. The gels were dried at RT, and protein digestion was performed in 10 mM ammonium bicarbonate containing trypsin (Thermo Scientific, Waltham, MA, USA) overnight at 37 °C. The peptides were extracted by adding 100% ACN to each tube and incubating for 15 min. The supernatant was transferred into a new microcentrifuge tube. All supernatants were dried using a centrifugal concentrator (TOMY, Tokyo, Japan). Samples were stored at −20 °C prior to mass spectrometric analysis [19].

### 2.5. Liquid Chromatography–Tandem Mass Spectrometry Analysis

Analysis of the digested peptides was performed using an Ultimate 3000 ultrahigh pressure system coupled with a SCIEX TripleTOF 6600+. Separation of the peptides was carried out in reversed-phase mode using a Pepmap100 C18 column (75 µm internal diameter × 150 mm length × 1.9 µm particle size) at 55 °C. Mobile phase A for liquid chromatography contained 0.05% formic acid (FA) in water, and mobile phase B contained 80% acetonitrile in 0.04% FA. After the samples were loaded into the column, the first linear gradient separation was operated for 95 min from 3 to 35% of mobile phase B at a flow rate of 300 nL/min. Regeneration of the column was performed with 90% mobile phase B for 10 min, followed by re-equilibration with 5% mobile phase B for 15 min. Precursor masses of 400–1500 m/z were collected within 250 ms under “high sensitivity” mode. Further fragmentation of each precursor spectrum was performed with a maximum of 30 precursors per cycle.

### 2.6. Data Analysis for Protein Identification

The MS spectra results recorded in wiff format were analyzed using Paragon™ Algorithm in ProteinPilot™ Software version 5.0.2 (Sciex, Concord, ON, Canada) installed with the Uniprot protein database of human (Taxonomy ID: 9606), retrieved from UniProtKB and assembled in FASTA format. The data were downloaded in August 2023, and the threshold of [Unused ProtScore (Conf)] ≥ 0.05 with 1% false discovery rate (FDR) was set to detect protein data with ≥10 peptides/protein.

### 2.7. ELISA for Plasma Fibrinogen Gamma Chain (FGG)

ELISA was performed to measure the plasma concentration of fibrinogen gamma chain in 18 plasma samples using a single-wash 90 min sandwich ELISA protocol provided by the manufacturer (Abcam, Cambridge, UK). Briefly, all plasma samples were subjected to a 2000× dilution in sample diluent, and 50 μL of either diluted plasma or the fibrinogen gamma chain standards (35–2300 pg/mL) were loaded into each well of the ELISA plate, along with 50 μL of the antibody cocktail into each well. Subsequently, the plate was sealed and incubated for an hour at RT on a plate shaker set to 400 rpm. After removing the liquid and performing a triple wash with 350 μL of the wash buffer, 100 μL of the TMB development solution was added and incubated in the dark for 15 min on a plate shaker set to 400 rpm. Each well received 100 μL stop solution, and the plate was shaken for a minute to mix prior to immediately recording the 450 nm absorbance. Each sample was run in duplicate, and the concentrations of plasma FGG between non-frailty and cognitive frailty were compared.

### 2.8. Statistical Analysis

Data are presented as mean ± standard deviation, and statistical analyses were executed using Graph Pad version 5.0. The Fisher’s exact test was used for categorical variables, and an unpaired *t*-test (two-tailed) was used for comparison of two sets of continuous variables. A *p*-value < 0.05 was considered statistically significant.

## 3. Results

### 3.1. Demographic Data of Participants

The summary details of the participants from each group are summarized in Table 1. It was found that there was a significant difference between groups in terms of age, weight, height, and MoCA score. The number of participants categorized by sex and educational background was still closely comparable.

### 3.2. 2-DE of Human Plasma Samples

**Figure 1:**
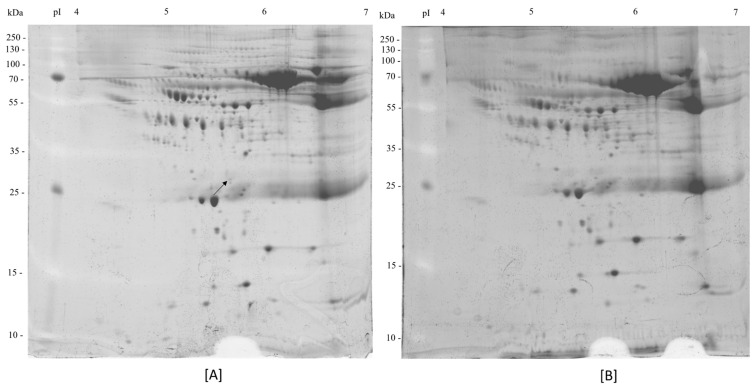
2-DE gel image profile. It was derived from non-frailty plasma (**A**) and cognitive frailty plasma samples (**B**), with IPG strip pH 4–7. The arrow indicates the protein identified in this study.

A comparison of differential protein expression profiles between plasma samples from non-frail and cognitively frail elderly individuals revealed distinct differences in protein patterns within the molecular weight range of 10 to 250 kDa (Figure 1). Notably, several protein spots within a narrow pH range showed significant variation, particularly at approximately 29 kDa, where the protein spot intensity in non-frail plasma was increased by approximately two-fold compared to cognitively frail plasma. Two additional protein candidates are presented in Table S1 of the Supplementary Materials.

### 3.3. Protein Identification

There were more than 100 spots shown on SYPRO^®^ Ruby-stained 2D gels in both non-frailty and cognitive frailty. In non-frailty, an overexpressed protein was spotted as compared to the cognitive frailty group. The spot was identified by SCIEX TripleTOF 6600+ system analysis. The details of the suggested protein identified by mass spectroscopy are shown in Table 2, the fibrinogen gamma chain (FGG), matched with UniProtKB ID of P02679 form FGG gene belonging to secreted localization. Details on biological processes, biological classes, and regulatory subclasses of the fibrinogen gamma chain are presented in Table 3.

### 3.4. Validation of FGG in Human Plasma by ELISA Analysis

**Figure 2:**
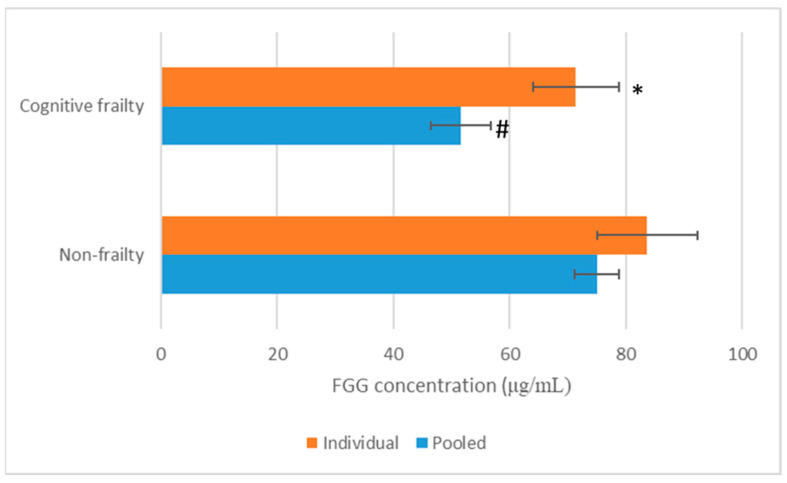
Concentrations of fibrinogen γ chain (FGG) determined by ELISA in plasma of cognitive frailty (*n* = 9) and non-frailty (*n* = 9) participants. Two determination methods were individual sample (Individual) and the pooled sample (Pooled) determinations. * and # indicate significant difference at *p* < 0.01 when compared with the similar determination method in non-frailty.

To validate whether the suggested protein was FGG protein, the expression levels of FGG in non-frailty and cognitive frailty elder plasma samples were measured using an ELISA kit. In plasma-pooled samples, the expression level of FGG in non-frailty was 75.05 ± 3.86 μg/mL, while it was 51.60 ± 5.14 μg/mL in cognitive frailty (Figure 2).

In addition, individual expression levels of the FGG chain in non-frailty and cognitive frailty plasma samples were also detected, and it was found that FGG in non-frailty (83.70 ± 8.69 μg/mL) was significantly higher (*p* < 0.01) than that in cognitive frailty (71.42 ± 7.45 μg/mL) (Figure 2).

## 4. Discussion

Cognitive frailty is a clinical condition that necessitates the development of reliable diagnostic tools for early detection because it is a silent harm leading to severe neurodegenerative diseases. Proteomic approaches offer a promising strategy for the identification of candidate biomarkers and the investigation of quantitative protein alterations in plasma and other biological specimens. In this study, we employed gel-based proteomic techniques, including two-dimensional electrophoresis (2-DE) and mass spectrometry (MS), to analyze and compare plasma protein profiles between cognitively frail and non-frail elderly.

We found that fibrinogen gamma (FGG) chain was markedly downregulated in cognitive frailty plasma samples compared with non-frailty elders. Our study showed that the plasma FGG chain level in non-frailty was more than 2.0-fold higher than in cognitive frailty, as observed by 2D gel electrophoresis. Secreted FGG was found in plasma of non-frailty at 83.70 (73.78–97.05) μg/mL, which was significantly higher than that in cognitive frailty measured at 71.42 (58.15–82.69) μg/mL.

FGG is a subunit of fibrinogen, which is a large complex glycoprotein with MW of 340 kDa containing α, β, and γ subunits encoded by *FGA*, *FGB*, and *FGG* genes, respectively [20]. The role of FGG in fibrinogen is to bind to the cell surface through integrin using its C-terminal region that plays a crucial role in thrombosis, angiogenesis, and inflammation [21,22]. Previous studies have reported that fibrinogenemia, characterized by significant deviations from the normal plasma fibrinogen concentration (2–4 mg/mL) [23,24,25], is frequently observed in patients with malignant tumors and is closely associated with tumor invasion, metastasis [26,27,28], angiogenesis [29], and tumor growth processes [30]. As fibrinogen is made of FGG, the reduction in FGG expression may affect the level of fibrinogen in plasma.

FGG is primarily synthesized and secreted into the bloodstream by the liver [31,32]. As a component of fibrinogen, FGG functions as a positive acute phase protein, with its plasma levels increasing during inflammation responses. FGG plays a critical role in coagulation, inflammation [33,34], and carcinogenesis [35,36] and has been proposed as a potential diagnostic marker in various diseases, including non-small-cell lung cancer [37] and bladder cancer [38]. Fibrinogen, a key element of the hemostatic system, is involved in both primary and secondary hemostatic responses. Upon thrombin-catalyzed cleavage of fibrinopeptides (Fp) A and B, fibrinogen is converted into fibrin, which spontaneously polymerizes into double-stranded protofibrils. These protofibrils further assemble into branched fibrin fibers, forming the fibrin clot [32,39]. Structurally, fibrinogen is a glycoprotein composed of three pairs of non-identical polypeptide chains (α, β, and γ), with FGG representing the γ-chain. The FGG gene also produces transcript variant isoforms via alternative splicing, including the FGG A precursor isoform [40].

Functionally, FGG is essential for fibrin polymerization and cross-linking, initiation of fibrinolysis, and regulation of factor XIII activity. It also mediates high-affinity binding to integrin expressed on platelets and leukocytes and facilitates thrombin binding to fibrin, exhibiting an inhibitory function formerly referred to as “anti-thrombin I” [41]. Frameshift mutations of FGG cause hypofibrinemia, a low serum fibrinogen level, which strongly indicates that FGG regulates fibrinogen secretion [42]. FGG has also been shown to inhibit platelet adhesion to fibrinogen via its interaction with hepatitis B splicing protein [43].

Dysregulation of FGG expression has been reported in multiple malignancies, including liver cancer [44], stomach cancer [23], and prostate cancer [45], suggesting its potential as a tumor-associated biomarker. Elevated FGG is related to gastric cancer [46], and plasma FGG levels have been proposed as predictors of prostate cancer progression [45]. Furthermore, FGG has been utilized to distinguish pancreatic cancer from normal serum samples, highlighting its potential clinical utility in cancer diagnostics [47]. Additionally, urine FGG levels could be used as a diagnostic marker for NSCLC [37]. Zhang et al. [48] found that FGG was highly expressed in the lung tissue of chronic obstructive pulmonary disease (COPD), and the expression level of FGG mRNA was related to the pulmonary function of patients. In addition, Zhang et al. [37] reported that FGG found in urinary proteins was a promising marker for non-small-cell lung cancer (NSCLC) due to the significant upregulation of FGG expression in 112 NSCLC patients detected by ELISA. Likewise, FGG was identified in the urine of IgA nephropathy (IgAN) patients using data independent acquisition (DIA)-based liquid chromatography–mass spectrometry (DIA-MS). Moreover, ELISA was also used in a validation cohort. The study suggested the potential use of urinary FGG as a non-invasive biomarker for the determination of mild renal fibrosis in IgAN [49].

Agnihotri et al. [50] conducted proteomic profiling of plasma samples obtained from 10 cognitively frail and 10 non-frail elderly individuals using two-dimensional gel electrophoresis (2-DE) combined with matrix-assisted laser desorption/ionization time-of-flight/time-of-flight mass spectrometry (MALDI-TOF/TOF). A total of 105 protein spots were identified. Among these, 22 spots in the frail group and 12 spots in the non-frail group exhibited differential expression. Mass spectrometric analysis revealed eight upregulated proteins in the frail group: haptoglobin (Hp) isoforms (1-1, 2-1, and 2-2), plasma amyloid A1, trafficking kinesin-binding protein 1 (TRAK1), nuclear body protein SP110, NOD-like receptor family CARD domain-containing protein 3 (NLRC3), matrix metalloproteinase-12 (MMP12), mortalin, and nucleoside diphosphate kinase 3 (NDK3). Additionally, five downregulated protein spots in the frail group were identified as PSRC1, NKG2A, and three unnamed protein products. Lin et al. [51] used proteomic approaches to identify frailty and non-frailty biomarkers in six older adults. It was found that 31 proteins, such as angiotensinogen (ANGT), kininogen-1 (KG), and antithrombin III (AT), in plasma were significantly elevated in frailty, while three proteins, Ig kappa chain V-III region WOL, COX7A2, and albumin, were significantly low. It was suggested that the levels of sestrin1 and sestrin2 in serum were significantly low and associated with frailty syndrome [52].

As cognitive frailty in elders could be worsened by the development of other neurodegenerative diseases, such as dementia and Alzheimer’s disease, if left untreated, proper treatments could reverse cognitive frailty back to normal cognition and or non-frailty. It is worth finding biomarkers to monitor the early development of these severe diseases, as the biomarkers may eventually shift to those specific for the condition. For example, amyloid-beta (Aβ) 42/Aβ40 ratio and phosphorylated tau derived from cerebrospinal fluid provide reliable indicators of Alzheimer’s disease, while emerging biomarkers like microtubule binding region (MTBR)-tau243 offer insights into disease progression. Likewise, matrix metalloproteinases, soluble platelet-derived growth factor receptor-β, and inflammatory markers reflect vascular cognitive impairment and dementia [53]. Moreover, the meta-analysis of various blood biomarkers associated with neuronal function as indicators of vascular cognitive impairment (VCI) suggested that amyloid beta 42 (Aβ42), amyloid beta 40 (Aβ40), Aβ42/Aβ40 ratio, neurofilament light (NfL), and S100B are significant biomarkers of VCI when compared with non-VCI [54].

## 5. Conclusions

This study employed proteomic approaches to differentially investigate the expression of protein markers in both non-frailty and cognitive frailty plasma samples from elders who resided in Saraphi District, Chiang Mai, Thailand. It was found that FGG chain was significantly downregulated in cognitive frailty plasma. In addition, ELISA was used to validate the expression level of FGG chain in both non-frailty and cognitive frailty. Cognitively frail elders had significantly lower levels of FGG chain in their plasmas when compared with non-frail elders. This study suggests that FGG chain may become one of the potential biomarkers used for early detection of the occurrence of cognitive frailty and to prevent the emergence of this deteriorated condition as well as for further diagnostic and therapeutic applications. However, further studies are still required for assurance in a larger study group or cohort and for understanding the mechanisms on how FGG is involved in cognitive frailty.

## Figures and Tables

**Table 1:** Summary of demographic information of the participants.

Parameters	Non-Frailty (*n* = 9)	Cognitive Frailty (*n* = 9)	*p*-Value
Age (years)	72.33 ± 5.34	69.67 ± 4.42	0.299
Weight (kg)	55.61 ± 8.92	60.06 ± 12.88	0.367
Height (cm)	157.22 ± 7.08	162.00 ± 8.46	0.293
Actual MoCA score (points)	19.11 ± 3.72	21.11 ± 2.93	0.067
Sex (male/female)	3/6	5/4	-
Educational level (Grade 1–3/Grade 4–6/High school or over)	6/1/2	8/0/1	-

**Table 2:** Protein identified from non-frail and cognitive frailty plasma samples by LC–MS/MS analysis.

Protein Spot	Protein Name	Accession No.^a^	Spot Location	Theoretical MW (kDa)	%Cov (95)	pI	Peptides Matched (95%)	Fold Change (FC)
Upregulation in non-frail								
1	Fibrinogen gamma chain	P02679	Non-frail	51.5	4.194	5.60	2	>2

^a^ Accession no. was obtained in UniProt database: http://www.uniprot.org/ (accessed on 10 June 2024).

**Table 3:** Biological details of the validated abundant protein.

Protein Identification Number	Protein Name (Gene)	Biological Roles	Biological Categories
P02679	Fibrinogen γ chain (*FGG*)	Many biological roles are suggested as follows: Platelet degranulation, including fibrinolysis, blood coagulation, fibrin clot formation, platelet aggregation, plasminogen activation, platelet activation Innate immune response Response to calcium ion Positive regulation of peptide hormone secretion Positive regulation of heterotypic cell–cell adhesion Involvement of protein polymerization in both cellular protein complex assembly and extracellular matrix (ECM) organization Positive regulation of exocytosis Negative regulation of endothelial cell apoptotic process Positive regulation of vasoconstriction. Positive regulation of substrate adhesion-dependent cell spreading Signaling pathway inhibition of death domain receptors for extrinsic apoptosis and induction of bacterial agglutination	Immune system, regulation, transport and homeostasis, coagulation, fibrinolysis, response, cell and ECM organization, protein metabolism

## Data Availability

Data are included in the article or Supplementary Materials; further inquiries can be directed to the corresponding author.

## Supplementary Materials

The following supporting information can be downloaded at: https://www.mdpi.com/article/10.3390/life15081330/s1. Table S1 List of candidate proteins differentially expressed between non-frailty and cognitive frailty elders and as identified by 2D-PAGE followed by LC-MS/MS.

## Abbreviations

The following abbreviations are used in this manuscript:

MCI	Mild cognitive impairment
MoCA-B	The Montreal Cognitive Assessment-Basic
FGG	Fibrinogen gamma chain
