# Supplementary material for: Proteomics Analysis of Plasma Biomarker of Cognitive Frailty in Elders Who Locally Reside in Chiang Mai Province of Thailand
Source: Life (Basel). 2025 Aug 21;15(8):1330. doi: 10.3390/life15081330 (PMC12387196; doi:10.3390/life15081330)
Supplement: Supplementary file 1 [file life-15-01330-s001.zip › life-3795810-supplementary.pdf]

Table S1. List of candidate proteins differentially expressed between non-frailty and cognitive frailty elders and as identified by 2D-PAGE, followed by LC–MS/MS

Protein spot	Protein name	Accession no.	Spot location	Theoretical MW (kDa)	pI	Fold Change (FC)
1	Fibrinogen gamma chain (FGG)	P02679	Non-frail	51.5	5.60	> 2
2	Small proline-rich protein 2A (SPRR2A)	P35326	Non-frail	7.965	6.85	>2
3	Complement C4-A (C4-A)	P0C0L4	Cognitive frailty	93	n.a.	>2

* n.a. = data are not available

We have verified two protein candidates, which were FGG and SPRR2A, using commercial ELISA kits. It was found that only FGG showed consistent results in the 2D-PAGE, followed by LC–MS/MS.
